# Normatively Irrelevant Affective Cues Affect Risk-Taking under Uncertainty: Insights from the Iowa Gambling Task (IGT), Skin Conductance Response, and Heart Rate Variability

**DOI:** 10.3390/brainsci11030336

**Published:** 2021-03-06

**Authors:** Giulia Priolo, Marco D’Alessandro, Andrea Bizzego, Nicolao Bonini

**Affiliations:** 1 Department of Psychology and Cognitive Sciences, University of Trento, 38068 Rovereto, Italy; andrea.bizzego@unitn.it; 2 Institute of Cognitive Sciences and Technologies, National Research Council, 00185 Rome, Italy; marco.dal92@gmail.com; 3 Department of Economics and Management, University of Trento, 38122 Trento, Italy; nicolao.bonini@unitn.it

**Keywords:** decision-making, behavioral economics, psychophysiology, risk-taking, skin conductance response, heart rate variability, IGT, somatic marker hypothesis

## Abstract

Being able to distinguish between safe and risky options is paramount in making functional choices. However, deliberate manipulation of decision-makers emotions can lead to risky behaviors. This study aims at understanding how affective reactions driven by normatively irrelevant affective cues can interfere with risk-taking. Good and Bad decks of the Iowa Gambling Task have been manipulated to make them unpleasant through a negative auditory manipulation. Anticipatory skin conductance response (SCR) and heart rate variability (HRV) have been investigated in line with the somatic marker hypothesis. Results showed fewer selections from Good decks when they were negatively manipulated (i.e., Incongruent condition). No effect of the manipulation was detected when Bad decks were negatively manipulated (i.e., Congruent condition). Higher anticipatory SCR was associated with Bad decks in Congruent condition. Slower heart rate was found before selections from Good decks in Control and Congruent condition and from Bad decks in Incongruent condition. Differences in heart rate between Bad and Good decks were also detected in Congruent condition. Results shed light on how normatively irrelevant affective cues can interfere with risk-taking.

## 1. Introduction

Being able to distinguish between safe and risky options is paramount to make functional choices in our daily lives. However, especially in a highly complex and uncertain world like the one we are living in, this is not always an easy task. 

Research has increasingly highlighted the role of emotional reactions in helping people to generate an adaptive response through a quicker and easier way to navigate complexity and uncertainty [1,2]. In the risk studies domain, for example, approaches such as the “*risk as feelings*” [3] assume that people base their judgments not only on what they think or know about an event/activity but also on what they feel about it (“*I like it/I dislike it”*). Following this theoretical approach, events/activities are marked in people’s minds with positive or negative affective reactions which occur rapidly and automatically and can be experienced, consciously or not, as a feeling state. Thus, according to the so-called “affect heuristic” [4,5], affective reactions come prior to and are used as an orienting mechanism in the decisional process and the perception of risks and benefits, especially in complex and uncertain situations. 

Similarly, the “somatic marker hypothesis” (SMH [6,7,8,9]) proposed that, in particular situations, bodily responses and corresponding central nervous representations, can generate emotional responses (“*somatic markers*”) that, in turn, can guide and assist the decisional process. This somatic mechanism is supposed to precede overt reasoning on declarative knowledge [10]. Indeed, somatic markers mark mental images of future outcomes with positive or negative emotional tags and are then used as a factor of efficiency and accuracy in decision making, even before conscious knowledge of the situation is available. The generation of these visceral tags can be detected from physiological signals (e.g., skin conductance response (SCR), heart rate variability) in terms of autonomic arousal. The ventromedial prefrontal cortex (vmPFC), orbital frontal cortex (OFC), and amygdala have been indicated as the neural basis and core brain regions of the somatic marker system [11,12,13]. Clinical observations on patients with damage to those areas have been indeed associated with decision deficits. Notably, this kind of patient performed poorly on the Iowa Gambling Task (IGT), an experimental paradigm used to simulate real-life decision-making under uncertainty [7,12,14]. The IGT is used as a tool to investigate the role of emotional reactions in the decisional process, as well as to test the SMH, in multiple fields [7,12,14,15]. The goal of the task is to maximize a $2000 virtual loan by picking cards from four decks. Two decks (A and B) are disadvantageous because they yield high immediate gains ($100) but high future unpredictable losses, i.e., overall net loss. We refer to them as “Bad decks”. The other two decks (C and D) are advantageous because they yield lower immediate gains (50$), but lower future unpredictable losses, i.e., overall net gain. We refer to them as “Good decks”. Participants can not guess the internal gambling schedule, and they do not know how many cards they will have to pick (the game is stopped after 100 selections), leaving them in an uncertain situation. Good performance at the IGT is typically associated with a higher proportion of selections from Good decks. 

Patients with brain damages, indeed, usually selected more cards from Bad decks rather than from Good ones and failed to produce anticipatory autonomic somatic response, measured as anticipatory skin conductance response, before selecting a card from a Bad deck. On the other hand, non-impaired control subjects gradually tended to avoid Bad decks and started to generate pronounced anticipatory SCR before selecting a card from those decks [7,14]. Specifically, “they begin to generate anticipatory SCR whenever they ponder a choice that turned out to be risky before they knew explicitly that it was a risky choice. The results suggest that, in normal individuals, nonconscious biases guide behavior before conscious knowledge does” [14]. Differently said, this affective reaction, experienced as a somatic state associated with the monetary outcome (deterministic gains and probable losses) of the decks, ultimately leads people with an intact somatic marker system to perform better, by helping them to distinguish between potentially risky choices (that are indeed avoided) and safer ones (that are indeed approached), even when their knowledge about the situation is not yet available or at least uncertain (“pre-hunch” period) [6,8,16]. 

In these terms, affective reactions can be considered beneficial to decision-making [6]. However, reliance on emotional reactions can also be detrimental. For example, induced strong and negative emotional states before the IGT have been associated with poorer performances [6,17,18,19]. 

The intuition that affective reactions, even if unrelated to structural aspects of an option (e.g., its outcome and chances), might disrupt decision-making is not new. For example, in the marketing and advertising domains, affective reactions are usually exploited to influence consumer choices through “normatively irrelevant affective cues”. With this term, we refer to aesthetic features (e.g., pictures, colors, or symbols) used to generate positive or negative affective reactions towards a product, but that do not inform the consumer about normative characteristics of the consumer good such as its price or quantitative or qualitative aspects [20,21,22,23]. Thus, from a rational and economic perspective, these aesthetic affective cues should be irrelevant in the options’ weight, while the consumer should focus only on their economic value to make his choice. 

Although the power of irrelevant affective cues is widely recognized in the consumer-behavior field, very few studies investigated their role in the domain of decision under uncertainty and risk-taking. A study from Gnambs and colleagues [24], for example, investigated the role of “normatively irrelevant” task-cues in the Balloon Analogue Risk Task (BART) [25]. The BART is typically used to study risk-taking in uncertain conditions. It has been found to correlate with everyday risky behavior such as drug consumption, smoking, and safety behaviors [25,26,27]. Gnambs and colleagues [24] found that the color of the balloon to be inflated (red vs. blue) affected participants’ risk-taking. Participants inflated the balloon less often when it was red than blue-colored (keeping constant the gambling schedule). The change in risk-taking behavior as a function of the color of the balloon was interpreted as due to the symbolic value of the red color, traditionally associated with danger.

Following this relatively uninvestigated research line, in our study, we aimed to demonstrate how the same normatively irrelevant affective cue could be both detrimental and beneficial to risk-taking behaviors in conditions of uncertainty. Specifically, we wanted to understand whether the affective reactions driven by a negative manipulation of the options (i.e., the hearing of an unpleasant sound associated with card selections) interfered with risk-taking in the IGT. 

Thus, in our study, a computerized manipulated version of the IGT was used (see Section 2.3.1). Specifically, the hearing of an unpleasant sound has been systematically associated with selections from the Bad decks in a first condition (Congruent condition) and to selections from Good decks in a second condition (Incongruent condition) to provoke a negatively valenced affective reaction (see Section 2.3.2). A condition with no manipulation was also included to serve as a control. The negative affective manipulation has been designed to be unrelated to the gains and losses contingency of the decks. Indeed, the unpleasant sound was heard each time participants selected a Bad or a Good deck, according to the condition, regardless of the monetary outcome of that selection. In these terms, the negative affective reaction driven by the unpleasant sound was normatively irrelevant to the overall task goal (i.e., maximize the profit), as it did not inform participants about the economic value of the decks. 

Therefore, in the experimental conditions, participants would have experienced two negative anticipatory affective reactions: the first affective reaction was driven by the deterministic hearing of the unpleasant sound associated with a certain type of deck and was irrelevant to the overall task goal, while the second affective reaction was driven by the possible negative monetary outcome of the decks and was thus relevant to the overall task goal. 

In line with the SMH, the relevant anticipatory affective reactions associated with the disadvantageous risk contingency of the Bad decks should have assisted participants’ decisional process and guided them to avoid those decks. However, we believed that the presence of another irrelevant affective reaction, driven by the unpleasant manipulation, would have influenced participants’ risk-taking. Specifically, we expected that in the Congruent condition, the irrelevant negative affective reaction driven by the unpleasant sound would have helped participants to avoid Bad decks, as this first irrelevant affective reaction would have been added to the relevant affective reaction associated with the probable disadvantageous outcome of those decks. Differently said, we expected Bad decks to be “more intensively” somatically marked. We hypothesized therefore that participants in the Congruent condition would have shown a higher proportion of selection from the Good decks compared to the control condition (H_1a_). From a physiological perspective, we expected to find higher autonomic activation (i.e., higher anticipatory SCR and higher heart rate) before selections from the Bad decks rather than before Good decks as suggested by the SMH. Indeed significant albeit small-to-medium associations between IGT performance and autonomic activation (measured as anticipatory SCR) have been found in a recent meta-analysis, alongside small differences preceding selections from Bad and Good decks [28]. However, due to the summative effect of the irrelevant and relevant affective reactions, we expected that autonomic activation before Bad decks in the Congruent condition would have been more pronounced compared to the control condition in which only the relevant affective reaction could have been experienced (H_1b_). 

Conversely, we expected that the irrelevant negative affective reaction would have been disruptive in the Incongruent condition. Indeed, in this condition, participants should have experienced an irrelevant negative affective reaction driven by the hearing of the unpleasant sound associated with the Good decks, and a relevant affective reaction associated with the disadvantageous contingency of the Bad decks. We believed that the presence of the irrelevant affective cue associated with the Good decks would have misled participants masking the advantageous probable outcome of those decks and leading them to choose a lower proportion of cards from the Good decks compared to the control condition (H_2a_). We also expected that this disruptive effect could have been detected also from a physiological point of view. Specifically, we expected to find a more pronounced autonomic activation before selections from the Good decks in the Incongruent condition rather than in the control condition (H_2b_). Indeed, when the Good decks were not manipulated as in the control condition, the generation of an alarming affective reaction was not necessary. However, in the Incongruent condition, the presence of the unpleasant sound associated with Good decks would have made those decks somatically marked, leading participants to generate a somatic anticipatory alarm reaction towards them that could have been used to guide their decisional process in the wrong direction.

Alongside classic anticipatory SCR, heart rate variability (HRV) was also included in the present study. The measurement of heart rate acceleration/deceleration is interesting because changes in HRV have been associated with autonomic activation [29,30,31,32,33]. In particular, a faster heart rate has been associated with heightened sympathetic arousal, reflecting preparation for defensive action, while heart rate deceleration has been associated with parasympathetic responses reflecting attentional orienting in response to a potential threat [34,35,36]. Neuroimaging studies also founded a link between HRV and brain regions (e.g., amygdala and vmPFC) involved in threat perception [32,33]. However, to the best of our knowledge, only few studies investigated both anticipatory SCR and HRV in relation to IGT performances, and results are mixed. A first study from Crone et al. [37] found good performance to be related to higher anticipatory SCR and heart rate slowing preceding selections from Bad decks (versus Good decks), but those results were not supported by a recent study from Hayes et al. [38]. Thus, our study aimed also to better investigate the effect of HRV in relation to IGT performance.

Finally, IGT performances have also been associated in the literature with some personality characteristics (impulsivity and sensation-seeking traits and thinking styles) and participants’ state mood at the moment of the task. Thus, possible effects of the aforementioned individual differences were controlled for also in the present study. Theoretical rationale, methods, and results can be found in the Supplementary Materials (Section 1, Tables S1 and S2). 

## 2. Materials and Methods

### 2.1. Participants

A total of one-hundred and forty-nine university students participated in the study. Students were enrolled through an online recruiting platform and received up to €10 as reimbursement for their participation. Two participants were excluded from the final sample for 1) failing to terminate the study and leaving before the end of the behavioral task or 2) declaring to already know the IGT.

Hence, the final sample comprised one-hundred and forty-seven participants (51% female, M_age_ = 22.22 years, SD = 2.79). Fifty of them were randomly assigned to the Control condition, forty-eight were assigned to the Congruent condition, and forty-eight to the Incongruent condition.

The study received approval from the local ethical committee (Research Ethics Committee of the University of Trento, protocol number: prot_2019_004), and ethical principles were followed under the Declaration of Helsinki. All participants provided their written informed consent to participate in the study.

### 2.2. Design and Procedure

The study design entailed three between-subject conditions based on decks’ manipulation: a “Control” condition in which the decks were not manipulated, i.e., IGT standard version with no sound played; a “Congruent” condition in which Bad decks were associated with the hearing of the unpleasant sound, while Good decks were silent; and an “Incongruent” condition in which Good decks were associated with the hearing of the unpleasant sound, while Bad decks were silent.

Data collection took place in a research laboratory in individual sessions of about 90 min. Participants were required not to drink coffee, tea, or other kinds of energy drinks nor to smoke for at least two hours before the experimental session to avoid comprised physiological data recording. Upon arrival at the laboratory, participants read and signed an informed consent form indicating that they would have participated in a research project about decisions in condition of uncertainty and that their electrodermal (EDA) and cardiac (ECG) activity would have been recorded. 

EDA and ECG electrodes were placed. Participants were instructed to remain as still as possible, not to move the hand with the electrodes, and not to cross their feet, legs, and arms during the whole data recording to avoid movement artifacts. Subsequently, physiological activity at rest was recorded for 5 min to represent the baseline condition. Participants were asked to relax and remain still while seating in a quiet room in front of a black screen. After the rest period, the IGT was administered according to the condition. To maintain participants’ engagement in the game, they were told that 10% of their final gain would have been added to a base fee of €5 up to a maximum of €10.

Once the task was over, they were debriefed and received their reimbursement.

### 2.3. Materials

#### 2.3.1. Computerized IGT

A computerized version of the Iowa Gambling Task was administered via an OpenSesame 3.2.8 program [39]. The original gain and losses schedule from Bechara et al. [7] was used. The instructions were adapted from Bechara et al. [12] and presented on screen. The game entailed 100 trials, after which it was automatically stopped. Each trial consisted of the following screens. (1) “Selection screen”: the four decks appeared on the screen in full color and were labeled as 1, 2, 3, and 4, representing, respectively, Bad and Good decks. The previous amount and the total amount gained were shown above the decks. Participants picked a card by pressing the corresponding number on the keyboard. No time constraints were given to make the choice. (2) “Response screen”: the chosen deck was shown highlighted in full color for 3 s, while the other decks appeared in the background in black and white. Meanwhile, the unpleasant sound was played via headphones according to the condition. (3) “Feedback screen”: the updated total and previous amount gained were shown for 4 s above the statements “you won € xxx” printed in green, and “you lost € xxx” printed in red, showing the gain and the possible loss for that card. (4) “Inter-trail interval (ITI) screen”: all the decks appeared in the background in black and white on the screen. No actions were allowed during this time, and the keyboard was blocked. This screen lasted for 6 s to let physiological indexes return to the baseline [11]. Then the next trial began again from the “Selection screen”. Participants performed two test trials to practice with the task and the interface before starting the proper game.

#### 2.3.2. Sound Manipulation

To obtain a negative affective manipulation of the decks, the hearing of an unpleasant sound was associated with card selection and played in the Response screen according to the experimental condition. The sound (Sound ID: 0378; Category: Daily Routine Sound; Description: Signal1) was selected from the extended version of the international catalog IADS-E [40] to have high arousal (M = 7.18, SD = 1.37) and low (i.e., negative) valence (M = 2.05, SD = 1.46) and tested in a pilot study (*N* = 14). Results confirmed the sound to be highly unpleasant and not related to any idea of danger or any other meaning.

#### 2.3.3. Physiological Indexes

Physiological signals of EDA and ECG have been recorded with a Biopac MP160 at a 2000 Hz sampling rate and processed with Python’s “pyphysio” library [41] for indexes extraction.

To quantify physiological arousal as a somatic marker in line with the SMH, we focused on anticipatory physiological reactions generated in the “pre-choice interval” of 5 s before each card selection, following Bechara et al. [11].

EDA signal has been collected through two reusable electrodes on the distal (first) phalanges of the index and middle fingers of the non-dominant hand of the participant. Each electrode was filled with isotonic gel before being applied. Participants were asked to wash their hands with neutral soap for hygienic reasons and to eliminate any traces of hand cream or other elements that could make the skin oily, resulting in poor skin conductance signal. EDA signals for each subject were manually inspected to detect noisy signals. Nine subjects have been excluded from the physiological analysis for having a too compromised signal. Anticipatory SCR was computed as the area under the detrended EDA curve in the pre-choice interval and expressed in amplitude units (µsec) per time interval (seconds). 

The ECG signal was recorded through three disposable electrodes applied on the chest following a LEAD-II placement (positive lead on the left rib, negative lead on the right clavicle, and ground lead on the right rib). Before attaching the electrodes, the site was cleaned with a mild abrasive gel. From the ECG signal, InterBeat Intervals (IBI, i.e., the distance between two consecutive heartbeats) have been extracted. IBI signals for each subject were then visually inspected for missing beats or misdetection and corrected or rejected (*N* = 13) as appropriate. Then, the difference between the AverageIBI (RRmean) in the pre-choice interval and the RRmean before the pre-choice interval has been computed (“DeltaRRmean”) as an index of HRV. In this case, positive DealtaRRmean values are associated with greater heart rate deceleration.

### 2.4. Statistical Analysis

The main purpose of the analysis was to test whether the normatively irrelevant affective cue affected risk-taking. Two separate analyses for behavioral and physiological data were performed. Specifically, a generalized mixed-effect model has been used to account for the effect of the experimental conditions on the probability to select from a Good deck as the IGT unfolded. Two separate linear mixed effect models have been used instead to investigate the effect of the experimental conditions on both anticipatory SCR and DeltaRRmean.

#### 2.4.1. Behavioral Data

A generalized linear mixed-effect model was used to investigate the relationship between the trial-by-trial unfolding of the task and the IGT performance. Task performance has been codified as the probability to select cards from the Good decks. Consistently, we adopted a logistic two-level multilevel model with subject as random effect to account for individual-level variability in task performance, as well as to test differences in task performance between conditions. The independent variable at the first level of the model consisted of the trial indicator (from 1 to 100). The dependent variable entailed a binary vector of responses codifying whether a Bad deck (coded as 0) or a Good deck (coded as 1) was selected in each trial. Regression parameters were estimated using maximum likelihood. Both random intercept and random slope were considered to obtain the maximally complex variance–covariance structure [42]. 

The analysis consisted of multiple phases of model comparisons. First, the overall data structure was examined to test the main effect of the “Trial” (e.g., the unfolding of the task) on the probability of selecting a Good deck with respect to a null model in which only the mean proportion of selections from Good decks was taken into account. The “Trial” predictor was rescaled by dividing it by the maximum number of trials, thus yielding a mapping from the original range [1,100] to [0.01,1]. Such a transformation ensured computational stability of the parameter estimation process. Then, both additional main effects of “Condition” (dummy coded) and interaction between “Trial” and “Condition” were considered to control for possible effect of the negative affective manipulation. The different models were compared by means of likelihood ratio tests (LRT), since all the models considered were nested, and the Akaike information criterion (AIC- [43]), which accounts for model’s complexity and parsimony.

Finally, coefficient estimates of the winning model in the log-odds scale were used to interpret the results.

#### 2.4.2. Physiological Data

Two separate linear mixed effect model analyses with subjects as random effect were performed to characterize the relationship between physiological signals (anticipatory SCR and DeltaRRmean) and the behavioral outcomes of selecting from Good or Bad decks along task blocks, as well as to test the effect of the negative manipulation. To include the temporal development of the IGT in the analyses, the task has been divided into four blocks [44]: block 1 (from trial 1 to 10), block 2 (from trial 11 to 20), block 3 (from trial 21 to 60), and block 4 (from trial 61 to 100). The different number of trials in each block ensured that enough Good and Bad decks selection datapoints were included in each block to allow analysis. Indeed, the probability of observing a Bad deck selection decreases in the last phases of the task. Individuals with no datapoints available for a Good or a Bad deck choice in one of the task blocks have been excluded from the analysis. Here, the type of selected Deck (Good or Bad), the experimental conditions, and Task Block have been considered as predictors and the physiological indexes (anticipatory SCR or DeltaRRmean) as dependent variable.

In both physiological analyses, we focused on the slope (fixed effect) codifying the increasing or decreasing trend of mean physiological indexes across task blocks. Differences between the physiological trends for Bad and Good decks in the three experimental conditions were taken into account by means of a paired-contrast analysis. 

The analyses were performed within the R statistical computing framework [45] with the aid of the lme4 package for fitting generalized linear mixed effect models [46]. Datasets of both behavioral and physiological analysis are deposited at: https://osf.io/up8mg/?view_only=dbb7cbb19e7c46108ca68ef7a4704548.

## 3. Results

### 3.1. Behavioral Analysis

Results showed a significant main effect of “Trial” since a significant reduction of the deviance could have been observed with respect to a null, baseline model (Chisq(1) = 74.076, *p* < 0.001). However, when the main effect of “Condition” was added to extend the model, the LRT showed a partial significance in the improvement of the model (Chisq(2) = 5.151, *p* = 0.076). This means that in general, individuals increased the chance to select Good decks as the task unfolded and that this improved performance could have been modulated by the different conditions. However, when the interaction term “Trial × Condition” was considered, no model improvements could have been detected (Chisq(2) = 1.532, *p* = 0.464). To further investigate whether the model with no interaction could have been a suitable one for the data, we considered the model’s complexity and used the AIC (Table 1).

As can be noticed, the third model, namely, the one accounting for both the effect of “Trial” and “Condition”, was selected as the best model. Together, the AIC and the LRT demonstrated that although individual performance improved as the IGT unfolded (which is a typical finding in the IGT with normal populations), the overall probability of selecting from a Good deck was modulated by the condition. Indeed, the lack of a significant interaction between the two independent variables revealed that the rate of change of the choice probability from Good decks did not differ between conditions. The coefficient estimates of the best model in the log-odds scale (Table 2) were considered to interpret the association between each predictor and the IGT performance.

**Figure 1:**
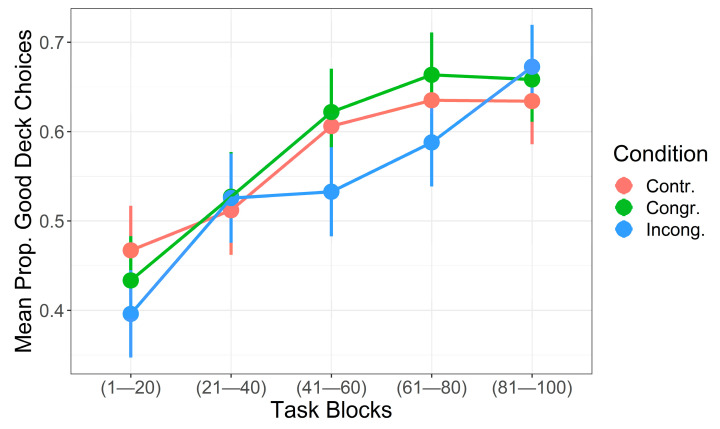
Descriptive statistics for the proportion of selections from Good decks across trails in the three experimental conditions. Error bars represent Standard Error.

**Figure 2:**
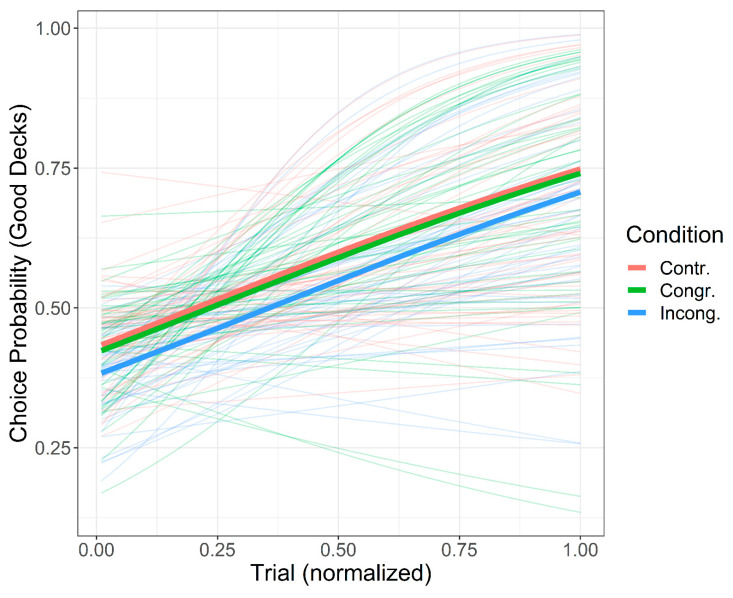
Probability of selecting Good decks as a function of the trial unfolding in the three experimental conditions. Strong and soft lines represent the probability of selecting Good decks at a group-level (fixed-effect) and at the individual-level (random-effects). Choice probabilities are reconstructed by applying an inverse transformation on the logits.

Since the model was characterized by the absence of the interaction term between “Trial” and “Condition”, the probability of selecting a Good deck as the task unfolded could be interpreted as a set of three parallel sub-models on the linear scale, one for each experimental condition (see Figure 1 for descriptive statistics for the behavioral data and Figure 2 for a comparison of the three sub-models on the probability scale). 

In general, the log-odds of selecting a Good deck increased by 0.0136 when moving from a given trial to the next one (note that here the regression coefficient was transformed back to the original predictor scale). In Congruent condition, no differences in the log-odds could have been observed through the evolution of the trial unfolding. Thus, the hypothesis that the probability to select cards from Good decks would have been higher in the Congruent condition compared to control (H_1a_) should be rejected. On the contrary, in the Incongruent condition, the log-odds of selecting Good decks decreased as accounted by the additive component “Condition (Incongruent)”, which equals −0.139. That is, the probability of choosing from a Good deck was systematically lower during the unfolding of the IGT in the Incongruent rather than in Congruent and Control conditions. This result supports the hypothesis that the probability to select cards from Good decks would have been lower in the Incongruent condition (H_2a_).

### 3.2. Physiological Analysis

#### 3.2.1. Skin Conductance Response

**Figure 3:**
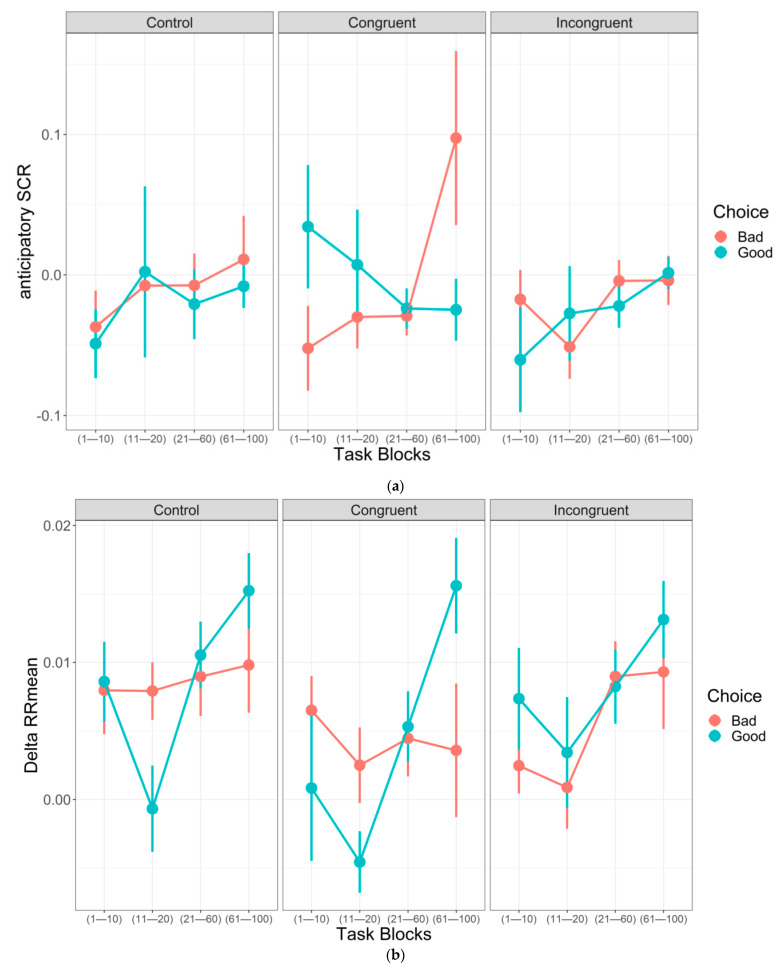
(**a**) Mean anticipatory SCR trend for Good decks and Bad decks for the three conditions as a function of Task Block; (**b**) DeltaRRmean trend for Good decks and Bad decks for the three conditions as a function of Task Block. Error bars represent Standard Error.

A linear mixed-effect model with anticipatory SCR as dependent variable and Deck, Condition, and Block as predictors was considered (see Supplementary Materials Section 2, Table S3 for the full model). Eighteen subjects were excluded from the analysis since they did not meet the inclusion criterion of having enough datapoints in at least one task block (Final *N* = 120). The model entailed the interaction Deck × Condition × Block. Results (Figure 3a and Table 3) showed that mean anticipatory SCR when selecting from Bad decks in the Congruent condition grew significantly as the task unfolded, thus confirming the hypothesis that higher physiological arousal would have been associated with Bad decks when they were negatively manipulated (H_1b_). No significant growing trend was detected for the other Deck × Condition combinations, thus suggesting rejecting the hypothesis that physiological arousal towards Good decks would have been higher when they were associated with the hearing of the unpleasant sound (H_2b_).

Results from a paired-contrast analysis (Table 4) showed a significant difference between the mean anticipatory SCR trend for Good and Bad decks in the Congruent condition (*t*(744) = 3.381, *p* < 0.01). Specifically, the anticipatory SCR trend before selections from Bad decks was higher than before selections from Good decks when Bad decks were associated with the hearing of the unpleasant sound, in line with H_1b_.

#### 3.2.2. Cardiac activity

A linear mixed-effect model with DeltaRRmean as dependent variable and Deck, Condition, and Block as predictors was considered (see Supplementary Materials Section 2, Table S4 for the full model). Seventeen subjects were excluded from the analysis since they did not meet the inclusion criterion of having enough datapoints in at least one task block (Final *N* = 117). The model entailed the interaction Deck × Condition × Block. Results (Figure 3b and Table 5) showed that DeltaRRmean for Good decks in Control condition and Congruent condition and for Bad decks in the Incongruent condition grew significantly as the task unfolded. No significant trend could have been detected for other Deck × Condition combinations.

Results from a paired-contrast analysis (Table 6) showed a significant difference between the DeltaRRmean trend for Bad decks and Good decks in the Congruent condition (*t*(696) = −3.064, *p* < 0.05). Specifically, DeltaRRmean trend increased more before selections from Good decks.

## 4. Discussion

In this study, we tested how the same normatively irrelevant affective cue could have been both beneficial and detrimental to risk-taking using a manipulated version of the IGT. Drawing on the SMH [6,7,8,9] and risk as feeling [3] approach, we hypothesized that an irrelevant affective cue (i.e., the hearing of an unpleasant sound) could have helped participants to choose even more advantageously when it was associated with selections from the Bad decks, while it would have misled participants to choose less advantageously when it was associated with Good decks. The normatively irrelevant affective cue was designed to be irrelevant to the overall goal task (i.e., maximize a monetary loan), as it was not informative about the monetary outcome of the decks. 

In the Congruent condition, we expected that the hearing of the unpleasant sound associated with selections from the Bad decks would have generated a normatively irrelevant affective reaction towards those decks that would have summed up with the relevant affective reaction driven by the disadvantageous risk contingency of the decks suggested by the SMH. We expected that this additive effect would have led participants to avoid even more selections from the Bad decks while preferring selections from the Good one in the Congruent condition compared to a control condition with no manipulation. However, no differences in the probability of selecting a Good deck were found between Congruent and Control condition. Furthermore, in both conditions, a significant increment in the probability to select from a Good deck as the task unfolded was detected. Said differently, in both conditions, participants initially sampled from all the decks and then increasingly tended to select more often from the Good ones, which is a typical finding with normal populations in the IGT [7,12,14,15]. 

From a physiological perspective, however, a higher trend in anticipatory SCR before selections from Bad decks was found in the Congruent condition compared to the control one as hypothesized. Thus, results suggest that the irrelevant affective reaction and the relevant somehow integrated to more intensively somatically mark Bad decks. However, as told above, this strengthened somatic marker did not affect participants’ behaviors towards safer performances. Moreover, no effects of anticipatory SCR were found in the Control condition. This result is in contrast with classic findings of SMH and with a recent metanalysis showing differences in anticipatory SCR between Bad and Good decks, even though those effects were small to medium [28]. 

Nevertheless, a peculiar effect of heart rate variability was detected. Indeed, a growing trend in heart rate deceleration was found before selections from the Good decks rather than before Bad decks in both Control and Congruent conditions. Furthermore, this trend was higher before selections from Good decks in Congruent condition. Previous literature associated anticipatory heart rate deceleration with attentional orienting responses in preparation for a possible threat [34,35,36]. Indeed, slower heart rate was found before selections from Bad decks and associated with better performance on the IGT [37]. However, a slower heart rate has been also associated with lower stress reaction due to the parasympathetic influence on the heart through the vagus nerve [29,30,31,33]. We can therefore speculate that participants in both Control and Congruent conditions were less stressed while pondering an advantageous choice. This would be in line with Damasio’s hypothesis that “when a positive somatic marker is juxtaposed to a particular future outcome it becomes a beacon of incentive” [7] to pursue that action. Indeed, our participants correctly chose more from Good decks in Control and Congruent condition and were less stressed before doing so (i.e., positive somatic marker) as shown by their cardiac activity. This might be the first empirical evidence of a positive somatic marker’s effect measured as heart rate variability, although more data are required to confirm this hypothesis.

Taken together, these results showed that the affective cue was effective in generating a different somatic response between Good and Bad decks when the latter were associated with it, but that it was ineffective from a behavioral point of view. We can speculate that the disadvantageous risk contingency of the Bad decks played a predominant role in guiding participants toward Good decks and that the effect of the affective cue has been actually irrelevant. These results are in line with some critiques raised to the SMH and IGT. For example, Maia and McCelland [47] suggested that the gains and losses contingency of the IGT might be more cognitively penetrable than suggested by the authors and that it is performed through access to explicit knowledge. This implies that the generation of somatic markers might be subsequent to conscious knowledge and not necessary for adequate performance. Moreover, learning models of the IGT, such as reinforcement learning models and reversal learning models, suggested that other mechanisms might be involved in IGT performance (see [48] for a review, see also [38,49]). 

In the Incongruent condition instead, we expected participants to experience a negative irrelevant affective reaction towards Good deck when they were associated with the hearing of the unpleasant sound and that this affective reaction would have had a disrupting effect on their performance. Results confirmed that participants in this condition showed a systematically lower probability to select from a Good deck compared to both Control and Congruent conditions, thus showing a higher risky behavior performing more potentially disadvantageous choices. The disrupting effect of the affective cue was also detected from a physiological point of view. Indeed, even though no difference between the decks could be detected for anticipatory SCR, a higher heart rate deceleration before selections from a Bad deck was found. We can speculate that the presence of the affective cue associated with the Good decks misled participants’ somatic marker system to associate a positive somatic marker with the riskier, but not negatively manipulated, Bad decks, making participants less stressed when pondering a potentially disadvantageous choice. Nevertheless, the affective cue did not seem to completely disrupt participants’ performances. Indeed, a growing trend in the probability to select a Good deck as the task unfolded was found as in the Control and Congruent condition. Said differently, their performance increasingly improved as the task unfolded, even though participants in this condition chose a lower amount of cards from Good decks. It seems therefore that participants in this condition adequately understood the differences in decks’ risk contingency (i.e., Bad decks alluring large gains but potentially larger losses, resulting in an overall net loss, and Good decks alluring smaller gains but also potentially smaller losses, resulting in an overall net gain) and learned they had to endure the hearing of the unpleasant sound to reach a larger final overall gain.

Taken together, these results seem to resemble real-life behaviors. Imagine being told by a doctor to avoid a particularly bad-tasting food you always disliked to get better health. In this situation, you probably will not have any trouble following the diet and improve your health. Similarly, participants in our study did not have trouble avoiding the Bad decks when they were associated with the negative affective cue. Imagine instead the opposite situation, in which the doctor recommends you to always eat the same bad-tasting food, as it is very beneficial for your health. In this situation, it is probable that you will stick with the diet as you want to have better health and will endure the unpleasant food to reach your goal. However, it is also probable that from time to time, you will not comply with the diet, preferring a delicious and not healthy food instead of the bad-tasting and healthy one. Similarly, participants in the Incongruent condition endured the unpleasant sound associated with the Good decks, as they recognized it was beneficial to their overall performance but had more trouble in doing so. 

### Limits and Future Directions

In this study, one negative auditory manipulation was used as an affective cue to make the decks unpleasant and elicit a normatively irrelevant affective reaction. Considering that the sound used was the same throughout the unfolding of the IGT, it is possible that participants got used to it, thus being less influenced by the manipulation, especially in the last portions of the game. Future studies could use two or more sounds to control for a possible habituation effect. Moreover, the manipulation used in this study was meant to be highly generically unpleasant. We did not focus on more specific and potentially powerful negative emotions, such as fear or disgust. It is possible that a manipulation able to elicit those specific categorial emotions could have led to stronger results. Indeed, fewer advantageous choices have been found in previous studies when Good decks were associated with fear-relevant stimuli (i.e., pictures of spiders and angry faces, respectively) in subjects with spider phobia or social anxiety [50,51,52]. In those studies, however, the stimuli used to manipulate the decks were emotionally relevant and meaningful for the participants. In our study instead, the affective cue was designed to elicit a general negative affective reaction of unpleasantness, not emotion-relevant or meaningful for participants. Future studies should investigate possible effects of affective cues able to generate categorial negative emotions such as fear or disgust while keeping the stimuli not meaningful to the participants.

Additionally, we did not test for the effect of pleasant affective cues. Nevertheless, it could be interesting to investigate if such cues could lead to similar results, but in the opposite direction, i.e., increased risky choices when they are affectively pleasant. 

Moreover, our negative manipulation represented a deterministic aversive outcome associated with the action of choosing a certain deck, but not with its monetary outcome. In these terms, the manipulation was irrelevant to the overall goal of the task (i.e., maximization of the initial monetary loan). However, it is possible that participants perceived the unpleasant sound as a punishment for their choice. This eventuality, in a reinforcement learning perspective [38,49], could have made the negative manipulation relevant to the task. Future studies should investigate this possibility.

In this study, we focused mainly on anticipatory physiological response according to the SMH, using SCR and HRV. However, some studies suggested that post-selection SCR (i.e., physiological activation in response to the choice’s outcome) might be more influential than anticipatory SCR on IGT performance [37,38,49,53,54,55]. Thus, the effect of post-selection arousal should be taken into consideration in future studies. Moreover, greater levels of HRV at rest have been related to better emotional regulation [32,56,57,58]. Future studies should investigate if the effect of the manipulation could be mediated by emotion regulation abilities. It could be that people who better regulate their emotional responses can be less influenced by the manipulation. Alongside resting HRV, emotional regulation abilities could also be investigated as a personality trait.

Finally, other types of physiological indexes such as pupil dilatation, respiration, or brain activity should be included in future studies to reach a clearer understanding of autonomic activation in response to anticipatory emotional reactions.

## 5. Conclusions

In this study, we showed how normatively irrelevant affective cues affect risk-taking in an uncertain situation using a manipulated version of the IGT. Participants’ choices in the Incongruent condition were systematically riskier than those of participants in the other two conditions (i.e., they systematically tended to select fewer cards from the Good decks). Moreover, their physiological reaction to riskier and safer options was disrupted. These findings show that the exploitation of affective reactions through the deliberative manipulation of affective cues of stimuli to guide decision-making can sometimes be misleading, as previously suggested [3,4,6]. It is therefore important to make decision-makers aware of this affective interference in their daily choices.

## Figures and Tables

**Table 1:** Model comparison’s results.

Model	Parameters	AIC	Deviance	Chisq.
Null Model	4	18,974.02	18,966	
Trial	5	18,902.95	18,892	74.076 (*p* < 0.001)
Trial + Condition	7	18,900.80	18,887	5.151 (*p* = 0.076)
Trial × Condition	9	18,903.26	18,885	1.532 (*p* = 0.464)

**Table 2:** Maximum likelihood estimates of fixed and random effect, *z*-values for regression coefficients, and variance of the random components of the best model.

Fixed Effects	Parameter	Estimate (SE)	*z*-Value	*p*-Value
	Intercept	−0.2807 (0.077)	−3.624	<0.001
	Trial	1.3609 (0.141)	9.704	<0.001
	Condition (Congr.)	−0.0414 (0.097)	−0.430	0.667
	Condition (Incongr.)	−0.2081 (0.096)	−2.166	0.03
**Random Effects**	**Parameter**	**Variance**	**Correlation**	
	Intercept	0.259		
	Trial (slope)	2.307	−0.65	

**Table 3:** Estimates and confidence intervals for the slopes of fixed effects of each Deck × Condition combination. A Kenward–Roger method has been used for degrees of freedom approximation.

Condition	Choice	Block. Trend	SE	*df*	Lower. CL	Upper. CL
Control	Bad	0.01443	0.0138	345	−0.01494	0.04124
**Congruent**	**Bad**	**0.04497**	**0.0138**	**345**	**0.01746**	**0.07249**
Incongruent	Bad	0.00875	0.0130	345	−0.01682	0.03429
Control	Good	0.00997	0.0138	345	−0.01745	0.03774
Congruent	Good	−0.02372	0.0138	345	−0.05081	0.00338
Incongruent	Good	0.01908	0.0130	345	−0.00649	0.04465

Bold: siginificative result.

**Table 4:** Results of paired-contrast analysis for each Deck × Condition combination. Approximated *p*-values have been computed with Tukey’s correction adjustment.

Contrast	Estimate	SE	*df*	*t*. Ratio	*p*-Value
Contr. Bad–Contr. Good	0.00445	0.0187	714	0.238	0.8117
**Congr. Bad–Congr. Good**	**0.06584**	**0.0187**	**714**	**3.523**	**0.0005**
Incong. Bad–Incong. Good	−0.01033	0.0174	714	−0.594	0.5522

Bold: siginificative result.

**Table 5:** Estimates and confidence intervals for the slopes of fixed effects of each Deck × Condition combination. A Kenward–Roger method has been used for degrees of freedom approximation.

Condition	Choice	Block. Trend	SE	*df*	Lower. CL	Upper. CL
Control	Bad	0.000659	0.00145	390	−0.002186	0.00350
Congruent	Bad	−0.000679	0.00141	390	−0.003450	0.00209
**Incongruent**	**Bad**	**0.002862**	**0.00137**	**390**	**0.000159**	**0.00556**
**Control**	**Good**	**0.003108**	**0.00145**	**390**	**0.000263**	**0.00595**
**Congruent**	**Good**	**0.005418**	**0.00141**	**390**	**0.002647**	**0.00819**
Incongruent	Good	0.002214	0.00137	390	−0.000489	0.00492

Bold: siginificative result.

**Table 6:** Results of paired-contrast analysis for each Deck × Condition combination. Approximated *p*-values have been computed with Tukey’s correction adjustment.

Contrast	Estimate	SE	*df*	*t*. Ratio	*p*-Value
Contr. Bad–Contr. Good	−0.002449	0.00204	696	−1.199	0.2310
**Congr. Bad–Congr. Good**	**−0.006097**	**0.00199**	**696**	**−3.064**	**0.0023**
Incong. Bad–Incong. Good	0.000648	0.00194	696	0.334	0.7385

## Data Availability

Publicly available datasets were analyzed in this study. This data can be found here: https://osf.io/up8mg/?view_only=dbb7cbb19e7c46108ca68ef7a4704548.

## Supplementary Materials

The following are available online at https://www.mdpi.com/2076-3425/11/3/336/s1: Section 1: Individual differences, Table S1: Model comparison’s results, Table S2: Maximum likelihood estimates of fixed and random effect, z-values for regression coefficients, and variance of the random components of the best model. Section 2: Full models for physiological data, Table S3: Full model for anticipatory SCR. Table S4: Full model for DeltaRRmean.
