# Supplementary material for: Normatively Irrelevant Affective Cues Affect Risk-Taking under Uncertainty: Insights from the Iowa Gambling Task (IGT), Skin Conductance Response, and Heart Rate Variability
Source: Brain Sci. 2021 Mar 6;11(3):336. doi: 10.3390/brainsci11030336 (PMC8001158; doi:10.3390/brainsci11030336)
Supplement: Supplementary file 1 [file brainsci-11-00336-s001.pdf]

Supplementary Materials

Section 1. Individual differences

1.1 Theoretical background

The role of some personality characteristics and state mood which have been related to IGT performance was also considered. Bechara and colleagues [1] suggested that some "cognitively disinhibited" nonclinical participants, i.e., those with high impulsivity traits, might express an impaired performance (i.e., more selections from Bad decks) on the IGT similar to vmPFC patients. This type of individuals, even in the absence of neurological deficits, is thought to share with patients a sort of "myopia for the future", i.e., a minimized attention toward positive and negative future consequences. Indeed, research [2,3] have found associations between riskier performances and impulsivity and sensation-seeking traits, measured with the BAS components of the BIS/BAS scales [4]. Accordingly, we expected that participants scoring high on impulsivity personality traits would have selected fewer cards from the Good decks in the IGT (H_1).

Risky performances in the IGT have also been associated with negative state mood at the moment of the task, in both clinical and non-clinical populations [2,3,5,6]. Similar results were found also when a negative state mood was induced prior to the task [7,8]. These results are in line with research associating negative affect to increased likelihood estimates for negative events and risk-taking [9–11]. Thus, in our study, we expected to find a negative correlation between negative state mood and selections from Good decks (H_2).

Little research has been conducted for both personality correlates and state mood's impact on IGT performance and results are not always consistent [2,3,5,12]. Thus, our study aimed also at adding insights on the role of both personal characteristics and state mood in the IGT performances.

Finally, a possible role of different thinking styles was also considered. Indeed, approaches such as the Dual-process theories [13–17] suggest that our mind can extract and process information according to two thinking styles. The *experiential* thinking style is automatic and based on intuitions and emotional reactions. The *analytic* thinking style (also called *rational*) instead, is deliberative and involved in more complex cognitive activities. We expected that participants who rely more on the experiential thinking style would have been more influenced by the normatively-irrelevant affective cue, compared to those who rely more on the analytic thinking style. Specifically, they would have selected fewer cards from the Bad decks when these decks were associated with the unpleasant sound (H_3). Conversely, they would have selected fewer cards from the Good decks when these decks were associated with the unpleasant sound (H_4).

1.2 Methods

To assess individual pre and post-test state mood an Italian translation of the PANAS-X Scale [18] was used. Participants were asked to rate from 1 (Very slightly or not at all) to 5 (Extremely) to what extent a series of words and phrases describing both positive and negative feelings and emotions correspond to their current state mood.

Impulsivity traits were measured with the Italian validated version of the BIS/BAS scale [4,19]. The questionnaire entails 20 items: 7 for the BIS scale and 13 for the BAS scale. The BAS scale is divided into three sub-scales: Drive, Reward Responsiveness, and Fun Seeking. The participants had to rate from 1 (Not at all) to 5 (Completely) how each item describes his/her person. Only the Fun Seeking subscale was considered for the analysis as an index of impulsive personality traits.

Participants' thinking style was assessed with the Italian validated version of the Rational-Experiential Multimodal Inventory (REIm-[20,21]). The questionnaire asks participants to rate from 1 (Completely false) to 5 (Completely true) if 42 items are true or false for themselves. The items are divided into two subscales, one for experiential thinking style and one for the analytic one.

1.3 Results

Starting from the best model, we assessed the effects of personality traits, with multiple additive models that were selected based on the AIC (Table 1 Main Text). Significant improvements with respect to the best model were detected for the inclusion of the BAS ($Chisq(1)=10.582$, $p<0.01$). When including also the PANAS subscales, a partially significant reduction in the deviance was observed ($Chisq(2)=5.956$, $p=0.051$). Differently,

when the REIM subscales were taken into account, no improvements could have been detected (Chisq(2)=0.422, p=0.809), thus refusing the hypotheses of possible effects of different thinking styles on IGT performance (H_3 and H_4). In sum, as an overall result, the model which included the PANAS subscales and the BAS was the best model when controlling for the effect of individual-level covariates (Table S1).

Table S1. Model comparison's results.

Model	Parameters	AIC	Deviance	Chisq.
Trial + Condition	7	18900.80	18887	
Trial + Condition + BAS	8	18892.21	18876	10.582 (p<0.01)
Trial + Condition + BAS + PANAS subscales	10	18890.26	18870	5.956 (p=0.508)
Trial + Condition + BAS + PANAS subscales + REIM subscales	12	18895.79	18870	0.422 (p=0.809)

The coefficient estimates of the best model in the log-odds scale (Table S2) were considered to interpret the association between each predictor and the IGT performance.

Table S2. Maximum likelihood estimates of fixed and random effect, z-values for regression coefficients, and variance of the random components of the best model.

Fixed Effects	Parameter	Estimate (SE)	z-value
	Intercept	-0.317 (0.07)	-4.197
	Trial	1.368 (0.14)	9.704
	Condition (Congr.)	-0.001 (0.09)	-0.020
	Condition (Incongr.)	-0.139 (0.09)	-1.478
	PANAS (Pos. Emo.)	0.001 (0.01)	0.234
	PANAS (Neg. Emo.)	-0.018 (0.01)	-2.462
	BAS (Fun Seek.)	-0.171 (0.05)	-3.427
**Random Effects**	**Parameter**	**Variance**	**Correlation**
	Intercept	0.251	
	Trial (slope)	2.302	-0.70

The negative coefficients of the PANAS subscale Negative Emotions and the Fun Seeking subscale indicated that as the test score increased the baseline probability to select Good decks decreased, confirming the hypotheses that disadvantageous performances in the IGT would have been positively associated with impulsivity traits (H_1) and negative state mood (H_2).

1.4 References

1. Bechara, A.; Tranel, D.; Damasio, H. Characterization of the Decision-Making Deficit of Patients with Ventromedial Prefrontal Cortex Lesions. *Brain* **2000**, *123*, 2189–2202, doi:10.1093/brain/123.11.2189.
2. Buelow, M.T.; Suhr, J.A. Construct Validity of the Iowa Gambling Task. *Neuropsychology Review* **2009**, *19*, 102–114, doi:10.1007/s11065-009-9083-4.
3. Suhr, J.A.; Tsanadis, J. Affect and Personality Correlates of the Iowa Gambling Task. *Personality and Individual Differences* **2007**, *43*, 27–36, doi:10.1016/j.paid.2006.11.004.
4. Carver, C.S.; White, T.L. Behavioral Inhibition, Behavioral Activation, and Affective Responses to Impending Reward and Punishment: The BIS/BAS Scales. *Journal of personality and social psychology* **1994**, *67*, 319–333, doi:10.1037/0022-3514.67.2.319.

5. Buelow, M.T.; Suhr, J.A. Personality Characteristics and State Mood Influence Individual Deck Selections on the Iowa Gambling Task. *Personality and Individual Differences* **2013**, *54*, 593–597, doi:10.1016/j.paid.2012.11.019.
6. Must, A.; Szabó, Z.; Bódi, N.; Szász, A.; Janka, Z.; Kéri, S. Sensitivity to Reward and Punishment and the Prefrontal Cortex in Major Depression. *Journal of Affective Disorders* **2006**, *90*, 209–215, doi:10.1016/j.jad.2005.12.005.
7. de Vries, M.; Holland, R.W.; Witteman, C.L.M. In the Winning Mood: Affect in the Iowa Gambling Task. *http://journal.sjdm.org/bb4.pdf* **2008**.
8. Shukla, M.; Rasmussen, E.C.; Nestor, P.G. Emotion and Decision-Making: Induced Mood Influences IGT Scores and Deck Selection Strategies. *Journal of Clinical and Experimental Neuropsychology* **2019**, *41*, 341–352, doi:10.1080/13803395.2018.1562049.
9. Johnson, E.J.; Tversky, A. Affect, Generalization, and the Perception of Risk. *Journal of Personality and Social Psychology* **1983**, *45*, 20–31, doi:10.1037/0022-3514.45.1.20.
10. Bruyneel, S.D.; Dewitte, S.; Franses, P.H.; Dekimpe, M.G. I Felt Low and My Purse Feels Light: Depleting Mood Regulation Attempts Affect Risk Decision Making. *Journal of Behavioral Decision Making* **2009**, *22*, 153–170, doi:https://doi.org/10.1002/bdm.619.
11. Mohanty, S.N.; Suar, D. Decision Making under Uncertainty and Information Processing in Positive and Negative Mood States. *Psychological Reports* **2014**, *115*, 91–105, doi:10.2466/20.04.PR0.115c16z2.
12. Suhr, J.; Hammers, D. Who Fails the Iowa Gambling Test (IGT)? Personality, Neuropsychological, and Near-Infrared Spectroscopy Findings in Healthy Young Controls. *Archives in Clinical Neuropsychology* **2010**, *25*, 293–302, doi:10.1093/arclin/acq017.
13. Epstein, S. Integration of the Cognitive and the Psychodynamic Unconscious. *American Psychologist* **1994**, 16.
14. Kahneman, D.; Frederick, S. Representativeness Revisited: Attribute Substitution in Intuitive Judgment. In *Heuristics and Biases*; Gilovich, T., Griffin, D., Kahneman, D., Eds.; Cambridge University Press, 2002; pp. 49–81 ISBN 978-0-521-79260-8.
15. Sloman, S.A. The Empirical Case for Two Systems of Reasoning. *Psychological Bulletin* **1996**, *119*, 3–22, doi:10.1037/0033-2909.119.1.3.
16. Sloman, S.A. Two systems of reasoning. In *Heuristics and biases: The psychology of intuitive judgment*; Cambridge University Press: New York, NY, US, 2002; pp. 379–396 ISBN 978-0-521-79260-8.
17. Stanovich, K.E.; West, R.F. Individual Differences in Reasoning: Implications for the Rationality Debate? *Behavioral and Brain Sciences* **2000**, *23*, 645–665, doi:10.1017/S0140525X00003435.
18. Watson, D.; Clark, L.A.; Tellegen, A. Development and Validation of Brief Measures of Positive and Negative Affect: The PANAS Scales. *Journal of Personality and Social Psychology* **1988**, *54*, 1063–1070, doi:10.1037//0022-3514.54.6.1063.
19. Leone, L.; Pierro, A.; Mannetti, L.; Leone, L. Validità Della Versione Italiana Delle Scale BIS/BAS Di Carver e White (1994): Generalizzabilità Della Struttura e Relazioni Con Costrutti Affini. *Gionale Italiano di Psicologia* **2002**, doi:10.1421/1245.
20. Norris, P.; Epstein, S. An Experiential Thinking Style: Its Facets and Relations With Objective and Subjective Criterion Measures. *Journal of Personality* **2011**, *79*, 1043–1080, doi:10.1111/j.1467-6494.2011.00718.x.
21. Monacis, L.; de Palo, V.; Di Nuovo, S.; Sinatra, M. Validation of the Rational and Experiential Multimodal Inventory in the Italian Context. *Psychological Reports* **2016**, *119*, 242–262, doi:10.1177/0033294116657623.

Section 2. Full models for physiological data

Table S.3 Full model for anticipatory SCR.

Fixed Effects	Parameter	Estimate (SE)	t-value	p-value
	Intercept	-0.027 (0.026)	-1.032	0.3027
	Task Block	0.012 (0.013)	0.882	0.3784
	Choice (Good)	-0.011 (0.033)	-0.346	0.7297

	Condition (Congr.)	-0.030 (0.037)	-0.792	0.4287
	Condition (Incongr.)	-0.007 (0.036)	-0.210	0.8336
	Task Block * Choice (Good)	0.001 (0.018)	0.027	0.9782
	Task Block * Condition (Congr.)	0.025 (0.019)	1.296	0.1958
	Task Block * Condition (Incongr.)	-0.002 (0.047)	-0.127	0.8992
	Choice (Good) * Condition (Congr.)	0.106 (0.018)	2.236	0.0256
	Choice (Adv.) * Condition (Incongr.)	-0.011 (0.046)	-0.247	0.8052
	Task Block * Choice (Good) * Condition (Congr.)	-0.061 (0.025)	-2.410	0.0162
	Task Block * Choice (Good) * Condition (Incongr.)	0.009 (0.024)	0.388	0.6984
**Random Effects**	**Parameter**	**Variance**	**Correlation**	
	Intercept	0.0059		
	Task Block (slope)	0.0011	-1.0	

Table S.4 Full model for DeltaRRmean.

Fixed Effects	Parameter	Estimate (SE)	t-value	p-value
	Intercept	0.007 (0.002)	2.699	0.0072
	Task Block	0.001 (0.001)	0.455	0.6490
	Choice (Good)	-0.003 (0.004)	-1.023	0.3064
	Condition (Congr.)	-0.002 (0.004)	-0.603	0.5468
	Condition (Incongr.)	-0.006 (0.004)	-1.674	0.0949
	Task Block * Choice (Good)	0.002 (0.002)	1.199	0.2309
	Task Block * Condition (Congr.)	-0.001 (0.002)	-0.662	0.5081
	Task Block * Condition (Incongr.)	-0.002 (0.001)	1.104	0.2699
	Choice (Good) * Condition (Congr.)	-0.005 (0.005)	-0.974	0.3301
	Choice (Good) * Condition (Incongr.)	0.007 (0.005)	1.425	0.1545
	Task Block * Choice (Good) * Condition (Congr.)	0.003 (0.003)	1.279	0.2012

	Task Block * Choice (Good) * Condition (Incongr.)	-0.003 (0.003)	-1.099	0.2720
**Random Effects**	**Parameter**	**Variance**	**Correlation**	
	Intercept	2.83E-05		
	Task Block (slope)	2.54E-07	-1.0	
